# The Efficiency of Color Space Channels to Quantify Color and Color Intensity Change in Liquids, pH Strips, and Lateral Flow Assays with Smartphones

**DOI:** 10.3390/s19235104

**Published:** 2019-11-21

**Authors:** Joost Laurus Dinant Nelis, Laszlo Bura, Yunfeng Zhao, Konstantin M. Burkin, Karen Rafferty, Christopher T. Elliott, Katrina Campbell

**Affiliations:** 1 Institute for Global Food Security, School of Biological Sciences, Queen’s University of Belfast, 19 Chlorine Gardens, Belfast BT9 5DL, UK; Y.Zhao@qub.ac.uk (Y.Z.); chris.elliott@qub.ac.uk (C.T.E.); 2 Department of Food and Drug, University of Parma, Parco Area delle Scienze 27/A, 43124 Parma, Italy; laszlo.bura@studenti.unipr.it; 3 School of Electronics, Electrical Engineering and Computer Science, Queen’s University Belfast, 125 Stranmillis Road, Belfast BT9 5AH, UK; K.Rafferty@ee.qub.ac.uk; 4 Faculty of Chemistry, Lomonosov Moscow State University, 1-3 Leninskiye Gory, GSP-1, Moscow 119991, Russia; burkin-kost@yandex.ru

**Keywords:** smartphone colorimetrics, lateral flow assay quantification, color space, image correction, food contaminant screening, allergens, background correction, point of site analyses

## Abstract

Bottom-up, end-user based feed, and food analysis through smartphone quantification of lateral flow assays (LFA) has the potential to cause a paradigm shift in testing capabilities. However, most developed devices do not test the presence of and implications of inter-phone variation. Much discussion remains regarding optimum color space for smartphone colorimetric analyses and, an in-depth comparison of color space performance is missing. Moreover, a light-shielding box is often used to avoid variations caused by background illumination while the use of such a bulky add-on may be avoidable through image background correction. Here, quantification performance of individual channels of RGB, HSV, and LAB color space and ΔRGB was determined for color and color intensity variation using pH strips, filter paper with dropped nanoparticles, and colored solutions. LAB and HSV color space channels never outperformed the best RGB channels in any test. Background correction avoided measurement variation if no direct sunlight was used and functioned more efficiently outside a light-shielding box (prediction errors < 5%/35% for color/color intensity change). The system was validated using various phones for quantification of major allergens (i.e., gluten in buffer, bovine milk in goat milk and goat cheese), and, pH in soil extracts with commercial pH strips and LFA. Inter-phone variation was significant for LFA quantification but low using pH strips (prediction errors < 10% for all six phones compared). Thus, assays based on color change hold the strongest promise for end-user adapted smartphone diagnostics.

## 1. Introduction

### 1.1. General Introduction

Detecting, quantifying, and mitigating against contamination in the food supply chain is paramount to global food security. High-end laboratory equipment such as mass spectrometry is often used for this purpose [1]. Unfortunately, such equipment is often unavailable in the developing world [2]. Moreover, contamination and fraud often go undetected due to a lack of surveillance. A recent report from the European Rapid Alert System for Food and Feed (RASFF) showed that alert notifications in 2017 increased 26% compared to 2016 and reported on various outbreaks (with pathogens and mycotoxins being most prominent) [3]. The main increase in alerts were follow up alerts representing additional testing on products that might have been placed on the market in another country [3]. This increase in follow up alerts might indicate that the system suffers from limited traceability in the industrialized global market, as has been reported previously [4,5]. Use of on-site detection methods (performed by primary producers, supermarkets, or even consumers) can complement the current systems and provide a means for developing countries to enhance food security. Indeed, a plethora of smartphone-based devices has been developed to complement laboratory-based analyses in several sectors including the food sector. Some examples are the fluorescent detection of antibodies against recombinant bovine growth hormone in milk [6,7], fluorescent detection of *Escherichia coli* in yoghurt and egg [8], colorimetric detection of marine toxins (okadaic acid and saxitoxin) in shellfish [9], aflatoxin B1 in maize [10], hazelnut allergen in cookies [11], and peanut allergen in cookies [12]. Such systems have great potential to influence the future market of food quality analyses particularly if analyses are rapid and straightforward allowing uptake at the consumer level. To this end, especially paper-based colorimetric assays such as the lateral flow assays have great potential since test results can be quantified rapidly with a smartphone [13]. A good example is the system reported by Ross et al., which enabled smartphone-based hazelnut allergen quantification within 2 min after applying the extract to the assay [11]. Other systems that may become rapid after optimization are liquid-based assays such as ELISA for which several examples exist of commercial diagnostic tests, such as various assays for mycotoxin analyses, that can be performed in under 10 min [14]. This being said, the food sector is relatively behind in implementation of smartphone-based technology for rapid/real time detection of contaminants when compared to other sectors such as medicine and environmental contaminant detection and it may be interesting to piggyback on such systems for food contaminant analyses [15].

### 1.2. Hyphenating Lateral Flow and Other Colorimetric Assays with Smartphones

A LFA is a paper-based platform consisting of a sample pad, a conjugate release pad, a membrane with a test and control line and an absorbent pad, all attached to a backing card. If a liquid sample containing a target is loaded onto the sample pad, it will run over the conjugate release pad and membrane to the absorbent pad by capillary force. In this process, the target can bind/form a complex with a labelled immunoreagent (often gold-nanoparticle, carbon black, or latex bead conjugated antibody) present in the conjugate release pad of the LFA. When this complex arrives at the test line other immunoreagents immobilized there (often antibodies in a sandwich assay set-up) can catch the complex which causes a colored line to appear. A control line is equally formed by a similar immunoreaction of other immunoreagents present in the conjugation pad which can complex specifically with other immunoreagents immobilized on the control line to ensure the assay functioned properly [16]. Thus, LFAs are rapid colorimetric tests with color intensity variation in relation to the target concentration and results can generally be read within 5–10 min [14]. Simply photographing such a test and quantifying the color by image analyses on a user friendly app with a smartphone without compromising the functionality of the phone is attractive [13]. Moreover, this combination has merit since smartphones are ubiquitous, allow for user independent quantification of the LFA and enable real-time and place stamped reporting of the results using the smartphone’s wireless connectivity and build-in GPS. Moreover, many LFA have already been commercialized for on-site, non-expert use for food contaminant screening [14]. However, only one commercial smartphone-based LFA reader (RIDA Smart app; R-Biopharm) was identified for food contaminant analyses from the > 300 commercial assays included in the mentioned database [14]. This assay quantifies various LFAs testing for mycotoxins but only works for those specific assays and on a few android-based smartphones. Evidently, consumer friendly rapid on-site analyses would greatly benefit from an app that is compatible with a large variety of phone-models and allows rapid quantification of a larger variety of (commercially available) colorimetric assays with phone A by interpolating on a calibration curve made in the laboratory with phone B. To this end, a universal approach for color quantification is needed. Color quantification with a smartphone using red green blue (RGB), hue saturation value (HSV), or lightness and chromatic axes A/B (LAB) color spaces have been reported for this purpose and were reviewed recently in the context of smartphone-based biosensors [17], smartphone-based food diagnostics [18], and quantitative LFA [19]. Roda et al. [17] outlines LAB and HSV color spaces as superior to RGB space for measuring small changes in color. However, which of the individual channels should be used or why HSV or LAB color spaces are better than RGB are not mentioned. Moreover, superior performance of the L channel of LAB is equally reported for the smartphone-based detection of hazelnut allergen with LFAs using carbon black based labeling [11] although no comparison with other channels was presented. In another work [20] paper-based detection of the mycotoxin producing black mold (*Stachybotrys chartarum*) is detected by using the R channel. However, no comparison with the B or G channel or other color spaces was reported. In these previous works [18,19] no recommendations on color space use were given although it has been reported that the R channel instead of the combined weighed RGB values lead to background reduction [21]. In another study, the performance of a ΔRGB or ΔLAB system was compared and ΔRGB outperformed ΔLAB for the quantification of plasmonic-ELISA assays [22] and found to perform well in a colorimetric paper-based assay [23]. To obtain Δ values a resultant of R, G, B or L, A, B vectors relative to control values was calculated [22]. However, no comparison between the single R, G, B or L, A, B channels with ΔRGB was performed in either study. Thus, there currently is no consensus regarding which color space/channel should be used for the smartphone image analyses needed for optimal functioning of smartphone hyphenated colorimetric assays such as ELISA, LFA, and other paper-based systems for food contaminant analyses. However, color space/channel choice clearly effects the performance of smartphone-based assays since several of the studies mentioned above reported substantial differences in assay performance in function of the color space/channel used. As a result, conversions to color spaces other than RGB are perhaps executed unnecessarily or suboptimal channels/color spaces may be chosen as a starting point for smartphone-based image analyses based on incomplete recommendations found in the literature. Other issues with current systems are (i) background illumination variation and (ii) inter-phone channel values variation. A light-shielding box is often used to tackle variation in background illumination [9,17,18,19], however, this detracts from the opportuneness of smartphone-based analyses if add-on items are required. Inter-phone channel value variation is especially important since it is undesirable to develop any system to be used by consumers if bespoke calibration is required for each phone. Unfortunately, most systems described previously were only tested with a single phone [9,11,17,18,19,20,24]. Overall, in the scientific literature there appears to be large variations in opinions regarding the use of color spaces and channel combinations. Moreover, no extensive comparison of the individual channels of RGB, LAB, HSV color space, and ΔRGB values with various phones has been identified.

### 1.3. Workflow Reported in This Study

In the present study, prediction accuracy of individual channels of RGB, HSV and LAB color space, and ΔRGB was determined for the quantification of color variation, using pH strips, and color intensity variation, using filter paper with dropped nanoparticles often used for LFA (i.e., gold, latex or carbon black nanoparticles) and nanoparticle and oxidized tetramethylbenzidine (TMB) solutions in ELISA wells. Background correction was shown to avoid measurement variation in the absence of direct sunlight more efficiently than a light-shielding box. Inter-phone (n = 6) variation was limited for color change quantification, permitting the quantification of color with phone A using a calibration curve constructed with phones B–F. The optimized system was validated using various phones with model food application exemplars in the quantification of gluten in buffer, bovine milk in goat milk and goat cheese, and pH determination in soil extracts with commercial pH strips and LFA.

## 2. Materials and Methods

### 2.1. Materials

Dibasic and monobasic sodium phosphate, sodium carbonate/bicarbonate, HCl (37%), 3,3′,5,5′-tetramethylbenzidine (TMB), NaOH, HAuCl_4_, sodium citrate, AgNO_3_, L-ascorbic acid, blue latex beads (LNPs), horseradish peroxidase (HRP), gluten from wheat (crude, ≥ 75% protein), and grade 1 Whatman filter paper were purchased from Sigma-Aldrich (Irvine, UK). Carbon black (N220) (CB) was obtained from Cabot Corporation (Ravenna, Italy), ZEU Proteon Gluten Express ZE/PR/GL25 and ZEU IC-BOVINO lateral flow assays for gluten and cow milk protein detection and ZEU Proteon ELISA were obtained from Zeulab (Zaragoza, Spain). Cow milk and goat cheese were purchased in the local market. Pure goat milk was home produced. DUS alkaline pH strips (5.00–8.50) were purchased from DFI (Gimhae, Korea). Soil was collected in Queen’s University garden (Belfast, UK). UV-Vis measurements were performed using a Tecan Safire IIplate reader. Smartphone measurements were performed with a Huawei P8 Lite (12 megapixels (MP)), iPhone 7 (12 MP), Samsung Galaxy Tab E (5 MP), Xiaomi mi5 (16 MP), HTC One M7 (4 MP) and a Samsung Galaxy J7 (13 MP), and a Smartscope Xscience (Ravensburger, The Netherlands) smartphone loop with 3D printed lenses for magnification where mentioned.

### 2.2. Nanoparticle Synthesis

Glassware was cleaned with piranha solution and aqua regia to remove all residues. The Turkevich method [25] was used for gold nanoparticle (GNP) synthesis. Briefly, 500 µL of 100 mM HAuCl_4_ together with 194.5 mL MQ was brought to boil, while stirring, in a round bottom flask equipped with a condenser. At boiling point, 5 ml sodium citrate solution (1% (w/v)), was added and the mixture was left boiling for 30 min then cooled down gradually. Stock GNP concentration (2.8 nM) was estimated using a protocol detailed in [26] and size estimations were reported elsewhere [27].

### 2.3. pH System (Color Change)

**Figure 1:**
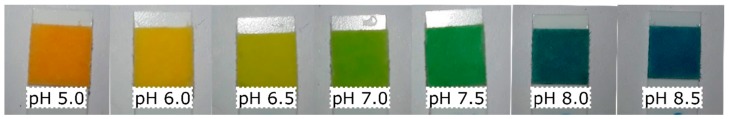
Exemplary images taken with the Huawei of pH strips that were used to build calibration curves.

Citrate-phosphate buffers (0.1 M; pH 5.0, 5.5), phosphate buffer (0.1 M; pH 6.0–7.5), and carbonate/bicarbonate buffers (0.1 M; pH 8.0, 8.5) were used for calibration (pH 5.0, 6.0, 6.5, 7.0, 7.5, 8.0, 8.5) and prediction (pH 5.5, 6.25, 6.75, 7.25, 7.75, 8.25) curves. For buffered soil extracts, 5 g of soil was added to 25 mL of buffer and vortexed. The pH was adjusted to match the buffer pH described above. Samples were allowed to settle 30 min and directly used for pH measurements. Images taken with the Huawei of pH strips dipped in various buffers are shown in Figure 1.

### 2.4. Nanoparticle Suspensions and Filter Paper Preparation

**Figure 2:**
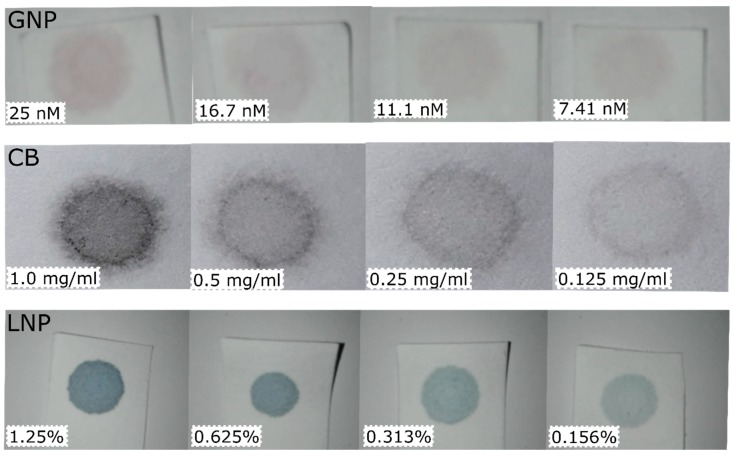
Exemplary images taken with the Huawei of the filter papers with nanomaterials on them at various concentrations. Top row are gold nanoparticles (GNP). Middle row are carbon black nanoparticles (CB). Bottom row are latex nanoparticles (LNP). Particle concentration of the solutions used is indicated in the left bottom corner of each image.

A 40X concentration was obtained from a stock GNP solution by centrifuging (13,000 RCF; 30 min; 20 °C). Concentrates were used to prepare a 2/3 dilution series from 37.5 up to 2.195 nM to construct calibration curves and a 2/3 dilution series from 31.25 up to 2.74 nM for predictions. For CB, a 10 mg/mL dispersion was sonicated 30 min then diluted to a 2 mg/mL dispersion and sonicated for another 10 min. Serial dilutions (2X) were made until 0.0078125 mg/mL. For predictions, a 1.5 mg/ml CB solution was made and diluted in 2X steps until 0.0117 mg/mL. LNP stock concentration (2.5%) was 2X diluted until 0.00977% to construct calibration curves. For the prediction set a 1.875% solution was diluted in 2X steps until 0.00732422%. Images taken with the Huawei of the nanomaterials on filter paper are shown in Figure 2.

### 2.5. Liquid Assays Preparation

**Figure 3:**
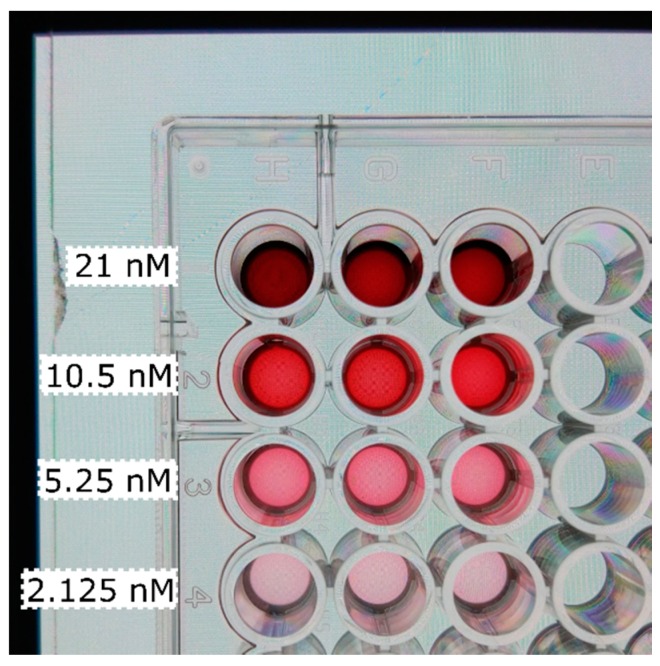
Image taken with the Xiaomi of colloid gold nanoparticles in a 96-well plate. Concentrations vary per row (as indicated). Each row (1–4) contains three replicas (H–F) used to construct the calibration curves. Backlight is provided using the white screen of the Huawei placed under the 96- well plate.

Colloid GNPs (200 µL) at varying concentration (8 step, 2X serial dilution from 84 nM for calibration and 7 step 2X dilution from 42 nM for prediction) or 150 µL of TMB with HRP at varying concentration (8 step 2X serial dilution from 60 pM for calibration and 6 step 2X dilution from 40 pM for prediction) were pipetted into transparent 96-well plates. TMB enzymatic reaction was stopped after 30 min with 4N H_2_SO_4_ (50 μL/well). Absorbance was read at 450 nm for plates with HRP and 513 nm (plasmon peak) for plates with colloid GNPs. Smartphone pictures were taken thereafter. An exemplary image taken with the Xiaomi of the colloid GNPs is shown in Figure 3.

### 2.6. Sample Preparation and Picture Capturing

**Figure 4:**
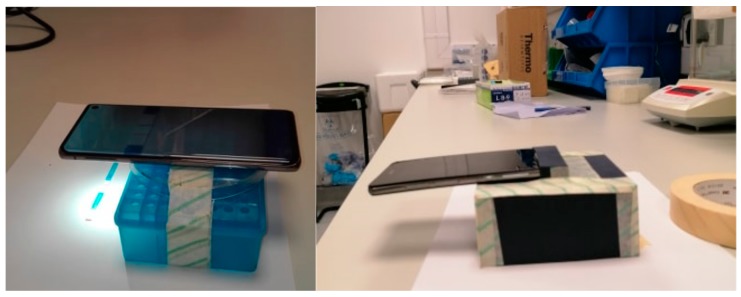
Left; an image of the Samsung taking an image of a LFA (the ZEU Proteon Gluten Express) under room-light conditions with the flashlight on at 5 cm distance. Right; an image of the light-shielding box used. The dimensions of the box are 11 × 11 × 5 cm to maintain the standard 5 cm as a distance for the photo capturing. The box had a hole in the center precisely for the camera and flash. The phone displayed on the box is the Huawei.

From GNP, CB, and LNP dilutions 5 μL was dropped on filter paper (n = 3), dried, and photographed. pH strips were immersed in buffer, dried, and photographed after 40 s. LFA test strips were photographed 10 min after exposure to extracts. For liquid assays, a phone with white screen was placed under the 96-well plate to provide counter illumination and avoid reflections in the images. All pictures were taken from 5 cm distance with the flashlight on (Figure 4). For background illumination experiments the following light changes were tried: Dark background in a closed windowless laboratory, normal room light (TL light), indirect sunlight in a windowsill, direct sunlight in a windowsill. A black cardboard box (11 × 11 × 5 cm) was made for the Huawei (Figure 4). The box had a hole precisely in the center for the camera and flash. Prediction images taken under varying background illumination were interpolated on calibration curves constructed at room light conditions to test robustness of the background correction applied. All images taken in the box for prediction were interpolated on a calibration curve constructed with images taken in the box at identical light conditions (room light).

### 2.7. Commercial Assays

**Figure 5:**
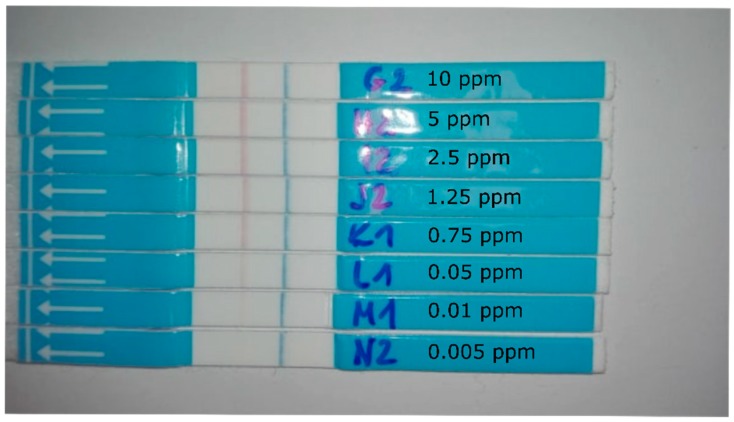
A set of LFAs used to build a calibration curve for gluten quantification with various smartphones. The concentrations of gluten used are indicated.

LFA for gluten, bovine milk, and cheese detection were used according to the instructions of the manufacturer. Briefly, (for estimation of gluten with the commercial LFA assay; ZEU Proteon Gluten Express ZE/PR/GL25), 1 g of gluten was extracted with 10 mL of the given extraction solution, vortexed and centrifuged for 10 min at 3500 RCF. The supernatant was diluted with analysis buffer to obtain 10, 5, 2.5, 1.25, 0.75, 0.5, 0.025, 0.01, and 0.005 ppm of gluten for calibration curve construction and 2, 0.5, 0.3, 0.15, 0.02, and 0.0075 ppm for predictions. The test strip was immersed in 250 µL of these dilutions for 10 min and photographed. For the estimation of cow milk/cheese in goat milk/cheese the ZEU-IC-BOVINO LFA assay was used. Cow milk was spiked into pure, home produced goat milk at 5%, 2.5%, 1.25%, 0.625%, 0.3125%, 0.15625%, 0.078125%, 0.04%, and 0.02% for calibration curve construction and at 3.75%, 1.875%, 0.46875%, 0.234375%, and 0.117188% for predictions. LFAs were immersed in two drops of these extracts diluted with three drops dilution solution. Purity of the goat cheese used was tested using the RC-BOVINO ELISA kit specific for the IgG of cow milk following the manufacturer’s instructions. Next extracts of cow cheese and pure goat cheese were prepared by adding 5 g of homogenized cheese to 10 ml H_2_O and vortexing. Extracts were centrifuged (10 min; 3000 RCF) and goat cheese supernatant was spiked with cow cheese supernatant (final concentrations 8%, 4%, 2%, 1%, 0.75%, 0.5%, and 0.1% of cow cheese) and used for calibration curve generation. Final concentrations of 6%, 3%, 1.5%, and 0.375% cow cheese were used for predictions. LFAs were immersed in one drop of extract diluted with two drops of the given dilution solution and photographed after 10 min. For all commercial assays duplicates were used. Figure 5 shows an image of a set of LFAs used to build a calibration curve for gluten quantification.

### 2.8. Scoring System

A scoring system was used to rate the performance of the channels which allowed to generate calibration curves with an R^2^ > 0.80 (R, G, B, V, L, H, and V). B of LAB (called C of LAC from hereon to avoid confusion with the B channel of RGB) was not considered since it performed only for pH and even there was outperformed by all other channels. R^2^ values on the regression functions fitted to the predicted concentrations were ranked from highest to lowest. Lowest being 1 point, highest 7 points. The same scoring system was used for the slopes of the regression fits. The channel with a slope closest to 1 got 7 points the next in line 6, etc. If no regression line could be fitted through the predictions the score was 0 points both for prediction and slope. Slope and R^2^ scores were summed up to get a final score for each channel.

### 2.9. Software and Data Treatment

Standard RGB values were detected with a free app from Google Play (RGB Android) (n = 3 per image) and converted to HSV and Cielab with the open source library of colormine.org (last accessed 11 August 2019). S values were rescaled to have a 1–100 range (instead of 0–1) to match RGB scale for visualization. For A and B values 128 points were added to allow a scale from 0–256 and avoid negative numbers complicating the background correction applied.

ΔRGB was calculated as specified in [22]. The applied image background correction was as follows:

Corrected channel values = (Raw signal value)/(Raw background value) × Raw signal value(1)

For paper-based assays white paper was used as the background except for the LFA where control lines were used instead. For liquid assays, wells filled with water adjacent to the tested wells were used as background. All channel values mentioned in the results are corrected channel values. Data analyses was performed with GraphPad Prism 6 Software. For post-hoc analyses, a one-way ANOVA Tukey’s multiple comparison test was used. For two-way ANOVA Sidak’s multiple comparison test was used. *p*-values were corrected for multiplicity. For calibration curves, a four-parameter dose-response curve was used unless mentioned otherwise. Mean average error percentile (MAE) was calculated on the totality of predictions over the concentration ranges mentioned above. LOD, IC_50_, and linear range were obtained by interpolating 90%, 50%, and 20%–80% signal values from fitted normalized curves.

## 3. Results and Discussion

### 3.1. Comparing Channel Performance on a Huawei P8

**Figure 6:**
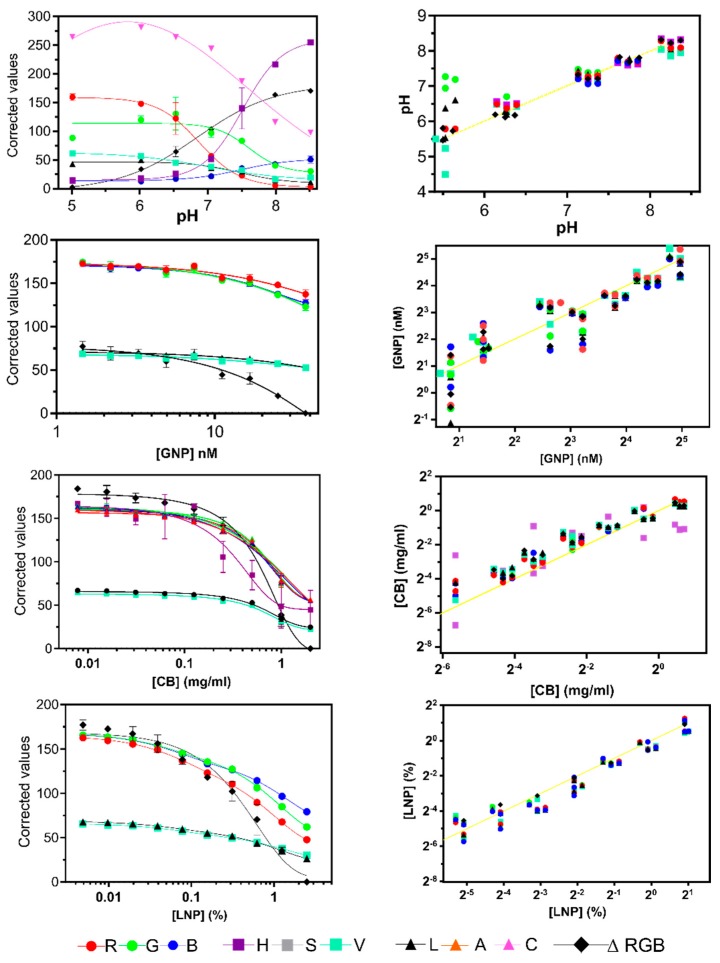
The left column shows calibration curves (with an R^2^ > 0.8) obtained from background corrected channel values from the RGB, HSV, and LAB (Called LAC to avoid confusion of the B channels of RGB and LAB) color spaces for various pH values, GNP, CB, and LNP concentrations. The right column shows scatter plots of predictions obtained with those calibration curves. The yellow line represents a perfect correlation (slope = 1). Color and symbol codes are indicated.

Various concentrations (n = 3) of GNP, CB, and LNP nanoparticles were dropped on filter paper and photographed. R, G, B, values of all pictures were extracted and used to calculate L, A, B, H, S, V and ΔRGB values. The values were fitted to either a four-parameter dose-response curve or a two- phase decay function, which ever resulted in the best fit in terms of R^2^ value (Table 1). The fitted calibration curves (with an R^2^ > 0.8) are depicted in Figure 6, left column. Of the LAB color space (called LAC to avoid confusion of B channels with RGB); only L generated adequate calibration curves in all color systems. A curve in all color systems had an R^2^ < 0.8 and were not used for predictions. C allowed generating a curve for pH determination only and symmetry was observed which made it only possible to predict pH below pH 7. Thus, C was not used for predictions. However, combining A and C might enable color change quantification, as shown previously [28]. From the HSV color space S never reached R^2^ values above 0.8 and was not used. H was effective in generating calibration curves for pH (as previously shown [29]) and CB, although errors were large in the latter color system with a mean average error (MAE) on all predictions (n = 18) of 90% ± 113%. The V channel worked well, producing calibration curves with R^2^ > 0.95 in all color systems except for GNP where R^2^ was 0.88. Prediction images of various pH, GNP, CB, and LNP concentrations were taken and interpolated using the fitted calibration curves of Figure 6. Predicted values are plotted in a scatter plot (Figure 6; right column). The R^2^ and slope values of the regressions are shown Table 2. For pH predictions, most channels shown in Figure 6 worked for predictions between pH 8.25 and 6.5. At the lowest pH tested (5.5) only R and ΔRGB allowed good predictions. For variation in nanoparticle concentrations low concentrations caused higher variance in all channels. H performed particularly badly in CB predictions. Larger variation in prediction was observed for the color intensity variations as for color change (MAE for the best channels were typically around 25%–35% ± 20%–40% and 1%–2.5% ± 1%–1.5%, respectively). See Section 3.2 for more detail and MAEs for various phones. This might be explained partly by the greater variance in the hand-made replicas for the latter system and partly by the potentially greater effects of background illumination variation on these tests (which was investigated in Section 3.3). To tease apart differences a scoring system (see Section 2.8) was adapted to rank the performance of the channels for each color system individually as well as for all color systems tested (Table 2 and Table 3). B, R, and ΔRGB scored the highest overall score, followed by V, L, G, and finally H. H underperformed since that channel was only effective in pH predictions. Moreover, in three out of four color systems either R, B, or ΔRGB had a top score (albeit shared with L in some cases, Table 2). For LNP predictions, the V channel scored highest. However, the R^2^ values and slopes of V, L, G, and B were close to each other (Table 2) and differences may only reflect random variation. In any case, this comparison shows that conversion to HSV and LAC color space did not increase performance compared to RGB in all color systems tested.

### 3.2. Comparing Channel Performance between Phones

**Figure 7:**
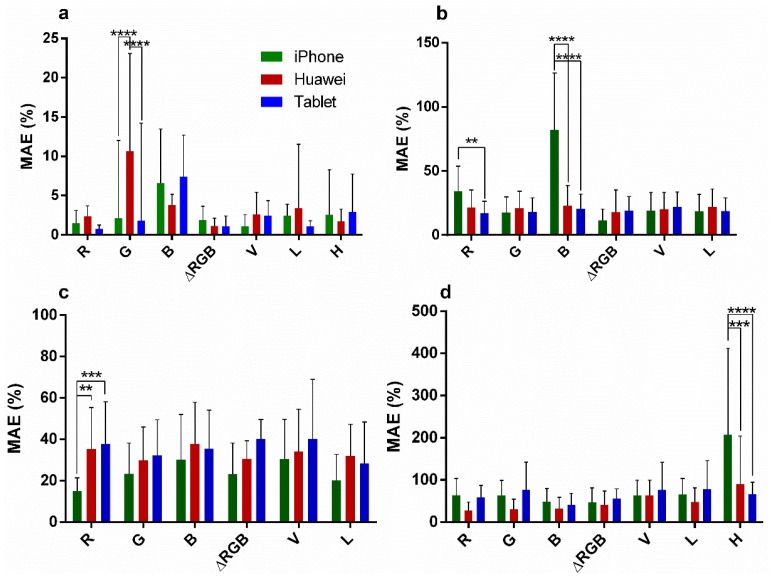
Mean average percentile errors (MAEs) for pH (**a**), (LNP) (**b**), (GNP) (**c**), and (CB) (**d**) predictions using an iPhone (dark green), Huawei (dark red), or Tablet (blue). Significant post-hoc Sidak multi comparisons of two-way ANOVAs are indicated. Stars indicate *p*-values with *p*-value correction for multiplicity. ** = *p* < 0.01, *** = *p* < 0.001, **** = *p* < 0.0001.

The experiments detailed in Section 3.1 were repeated on a tablet and iPhone to test if channel performance behaved similarly over various digital camera devices. Figure 7 shows a comparison of the total mean average percentile error (MAE) for the predictions (pH n = 15; GNP, LNP, and CB n = 18) over various concentrations. MAEs for pH calculations were low and stayed below 2.5% for R and ΔRGB for all phone models. For color intensity, low-end MAEs were typically 20%–30% ± 20%–30% for GNP and LNP predictions. For CB higher MAEs were observed although best functioning channels (B and ΔRGB) showed MAEs below 50%. Two-way ANOVAs (Table 4) show that for pH the variance in MAE caused by channels was significant (*p* = 0.0002), as well as interaction between channels and phone models (*p* < 0.006) although phone models alone did not cause significant variance in MAE (*p* = 0.24). For (LNP), (GNP), and (CB) predictions the phone model caused significant differences (*p* < 0.0001) as well as channel and interaction (*p* < 0.0001) in all but the GNP dataset, where only the phone model effects on MAEs was significant (*p* < 0.0001). Thus, choosing any specific channel as universally ideal for smartphone colorimetric analyses or even for a color specific system seems challenging, especially for color intensity change. Nonetheless, Sidak’s post-hoc simple effects within rows of multiple comparisons showed that most of the different effects between phones was limited to one or another channel of the RGB color system, or the H channel (for CB predictions). For ΔRGB no significant effects were observed in this test and thus shows that an error in one of the RGB channels can be compensated in this model, which makes it an interesting option. L and V equally show little variation between phones. However, variance on the MAE as well as absolute errors in the L and V values, although not significant, was larger when compared to the best functioning channel of RGB or ΔRGB in each color system (for L in pH predictions the highest MAE is 3.8% ± 8.4% versus 1.8% ± 1.7% for ΔRGB and for CB predictions the MAE was approximately 40%–50% ± 30% for B and ΔRGB and 50%–80% ± 30%–70% for L and V). Overall, H channel performed poorly in the color intensity experiments and did not outperform R, G, B, V, or L channels for color change predictions. Thus, H was no longer used in the following experiments.

### 3.3. Comparing Box/No-Box Effects on Predictions in Various Background Settings

**Figure 8:**
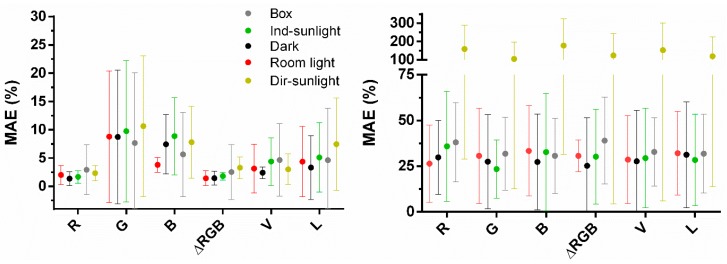
Left, MAEs for pH predictions (n = 15), right, MAEs for [GNP] predictions (n = 18), at various illumination conditions for R, G, B, ΔRGB, V, and L channels without use of a box and in a box at room illumination conditions. Images under dark (black balls) background were taken in a dark room; room background (red balls) in a windowless laboratory illuminated with a tube light bulb; indirect sunlight (green balls) in a windowsill at a cloudy day; direct sunlight (dark yellow balls) in a windowsill in full sunlight.

The effectiveness of the internal background correction was tested, with a Huawei, by comparing MAEs for pH and [GNP] prediction under varying background illumination with predictions when a light shielding box was used. Channels R, G, B, ΔRGB, V, and L were used since H has proven only functional for pH measurements and even for that application, performance was suboptimal. Little variation in MAEs was observed for pH estimation using the channels R and ΔRGB, even at illumination in direct sunlight (Figure 8). Moreover, two-way ANOVA was only significant for channels and not for interaction or background illumination (Table 5). Interestingly, for R and ΔRGB predictions variation was bigger using the box compared to all background illumination conditions. This may be explained by extensive scatter and unequal light distribution within the box compared to when no box was used. If the background varies between two images but stays equal throughout the individual images then internal background correction should largely correct for it. However, the error can be introduced if background illumination within a picture is patchy. This may also explain the observation by Masawat et al. that a larger box produces less error on predictions as a small one [24]. For [GNP] predictions, no difference was observed between box and no box over the conditions dark, room light, and indirect sunlight for all channels. However, in direct sunlight the background correction applied ceases to function and two-way ANOVA and post-hoc multiple comparisons were highly significant (*p* < 0.0001) for background illumination and direct sunlight, respectively (Table 5). At all other background illumination conditions, the MAE and variation on the MAE were similar in all conditions, including using the box. Thus, the simple background illumination correction applied eliminates the necessity to use a box for all color systems tested if measuring in direct sunlight is avoided.

### 3.4. Channel Performance for Colored Liquids

**Figure 9:**
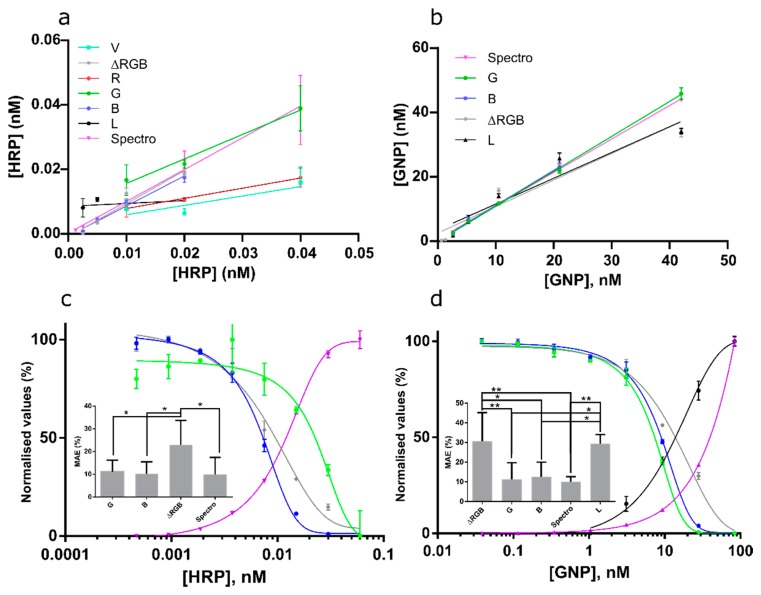
(**a**) Linear regression functions for oxidation of tetramethylbenzidine (TMB) with horseradish peroxidase (HRP) in ELISA wells for the smartphone analyses using various channels and a benchtop ELISA plate spectrometer. (**b**) Linear regression functions for colloid GNP at various concentrations in ELISA wells for the smartphone analyses using various channels and a benchtop ELISA plate spectrometer. Color codes and channels used are indicated. (**c**,**d**) normalized four-parameter dose-response curve fits for HRP oxidation of TMB (*a*) and colloid GNP (*b*) in ELISA wells. Green and blue circles stand for G and B channels, grey diamonds ΔRGB, magenta triangles absorption values measured by a spectrometer. (**c**,**d** inset) MAEs calculated from the predictions shown in (**a**,**b**). Stars indicate *p*-values from post-hoc analyses with *p*-value correction for multiplicity. * = *p* < 0.05, ** = *p* < 0.01.

Channel performance to quantify color intensity in liquid solutions (as typically done for ELISA) was investigated using various concentrations of colloid GNPs (mimicking plasmonic ELISA) and various amounts of HRP to oxidize TMB (mimicking standard HRP-based ELISA). Color change was measured using a benchtop spectrometer and a smartphone (Xiaomi) using R, G, B, ΔRGB, V, and L channels. All four-parameter dose response curves showed good fits (R^2^ > 0.8) and were used to predict GNP and HRP concentrations. Predictions and linear regressions are shown in Figure 9a,b. Slopes and R^2^ values of the calibration curves and R^2^ and slope values of the linear regression functions are shown in Table 6. For [HRP] prediction, the spectrometer, B, G, and ΔRGB channels were acceptable. For [GNP] prediction the spectrometer and G, B, ΔRGB, and L channels were acceptable (Figure 9a; Table 6). For comparison, the calibration curves of these channels and the spectrometer were normalized (Figure 9c,d). LOD, linear range, and IC_50_ for both assays are shown in Table 6. Interestingly, G, B, and ΔRGB channels for colloid [GNP] had a ~3X lower LOD as the absorption curve of the spectrometer (~1.5 versus ~7.5 nM, respectively). For the [HRP] curves, B and ΔRGB channels gave lower LODs as the spectrometer (~2.3 versus ~3.4 pM, respectively). Thus, the smartphone-based system was slightly more sensitive than the spectrometer. However, the linear range was reduced compared to the spectrometer. MAEs were calculated for G, B, ΔRGB, and spectrometer [HRP] predictions. For G and B only three concentrations (n = 3 × 3) were used since the linear range was reduced. For ΔRGB and spectrometer four concentrations (n = 4 × 3) were used. One-way ANOVA showed that MAEs for [HRP] prediction using G and B channels did not significantly differ from the MAEs for [HRP] prediction when the spectrometer was used (although with reduced range). ΔRGB had slightly higher MAEs (*p* < 0.05 Tukey post-hoc; Figure 9c inset). This is probably due to the error introduced into ΔRGB from the R channel which varied significantly and could not be used to build a calibration curve. For [GNP] predictions ΔRGB and L channels had significantly higher MAEs as G and B and the spectrometer (*p* < 0.01; Tukey post-hoc; Figure 9d inset). Again, the R channel could not be used to build a calibration curve.

### 3.5. Channel Performance Comparison Using Commercial LFA for Milk Allergen Detection

**Figure 10:**
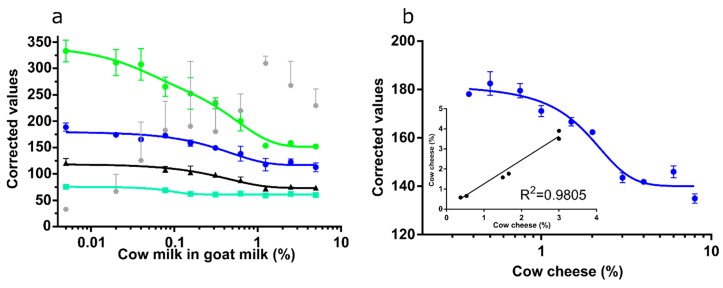
(**a**) G (green balls), B (blue balls), L (black triangles), and V (turquoise squares) channel values were fitted to a calibration curve for LFA quantification of cow milk spiked into pure goat milk. ΔRGB values (grey diamonds) did not allow to fit to a curve. (**b**) B channel values fitted to a calibration curve and, (inset), linear regression on predictions for cow milk in goat milk at three concentrations (n = 6).

**Figure 11:**
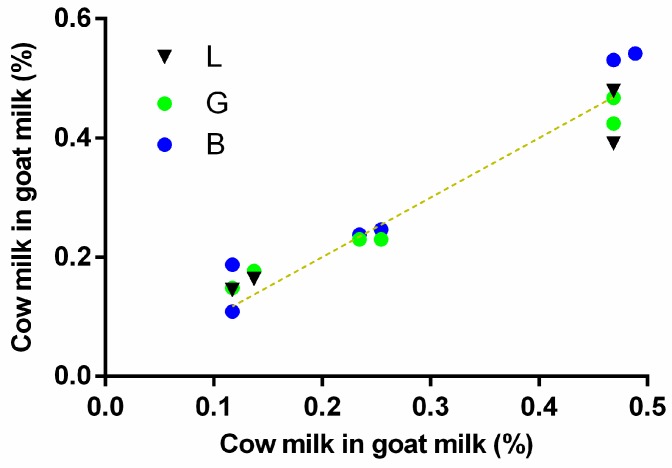
Predictions of cow milk (*x*-axis) in goat milk using L, G, and B channels. Each replica (n = 2) is indicated. Dashed line represents a curve at 45 degrees.

Commercial LFA for the quantification of bovine milk in goat milk and cow cheese in goat cheese (Figure 10a,b) were used to further challenge the smartphone-based quantification using the Huawei. For the LFA used to quantify bovine milk in goat milk (Figure 10a) G, B, L, and V channels showed promising calibration curves (R^2^ > 0.9). However, the curve determined with the V channel values was flat (min–max difference about 25 corrected value units). Equally, the L channel was quite flat and had slightly lower R^2^ values (0.91) as B and G (0.93 and 0.94, respectively). ΔRGB did not allow construction of a calibration curve, which was most likely caused by variations observed in the R channel values. Thus, only B, G, and L curves were used for predictions. Linear regressions on predictions were good for each channel (R^2^ > 0.95). However, the L channel did not predict at 0.23% cow milk (Figure 11). Goat cheese extract was spiked with cow cheese and used to test the applicability of the assay for the identification of cow protein in goat cheese. Here, only the B channel gave a satisfactory calibration curve (R^2^ = 0.951; Figure 10b). Predictions showed excellent linear regression (R^2^ > 0.98) (Figure 10b, inset).

### 3.6. Channel Performance Comparison Using Commercial LFA for Gluten and pH Strips for Soil pH Prediction

**Figure 12:**
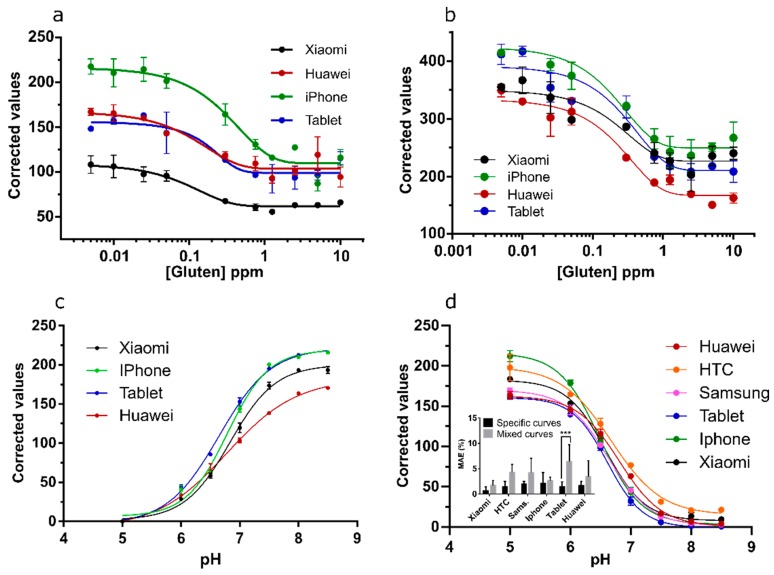
(**a**) B channel values fitted to four calibration curves for LFA quantification of gluten spiked into buffer with four phones (models indicated). (**b**) Calibration curves for LFA quantification using the smartscope and corrected G values. (**c**) Calibration curves for pH in buffered soil extract using corrected ΔRGB values. (**d**) R channel values fitted to calibration curves for pH estimation of buffered soil extracts with six phones (models indicated). Inset, the results of post-hoc analyses on the MAEs for pH predictions (n = 15) using the phone’s specific calibration curve or a mixed curve of all phone models except the one used for prediction. Stars indicate *p*-values with *p*-value correction for multiplicity. *** = *p* < 0.001. All calibration curves were made using a four-parameter dose-response function.

Next, the ability to use various phones to quantify gluten with a commercial LFA, as well as pH strip quantification in buffered soil was tested (Figure 12). B values performed again optimally for the LFA quantification and were used to construct calibration curves for the quantification of gluten in a buffer using four different phones (Xiaomi, Huawei, iPhone, and Tablet) (Figure 12a). All phones enabled the construction of calibration curves (R^2^ = 0.93; 0.88; 0.94; 0.86 for Xiaomi, Huawei, iPhone, and Tablet, respectively). Unfortunately, the curves did not overlap, except for the Tablet and Huawei, and cannot be directly used by end-users without calibration of individual phones. Magnification of LFA strips was attempted to correct inter-phone variation. LFA were photographed under the lens of a low-cost instrument (the smartscope; Ravensburger). Here, calibration curves with G channel values showed the highest R^2^ values (0.87, 0.95, 0.93, and 0.92 for Xiaomi, Huawei, iPhone, and Tablet, respectively) (Figure 12b). Although the distance between the curves was narrower as in the previous set-up, the overlap of all curves was insufficient for the use of various phones to predict values on the same calibration curve. Thus, for color intensity variation using LFA, it seems that two- point calibration or camera calibration is necessary. Some suggested methods exist to obtain such calibration and improve sensitivity [30,31,32,33]. One interesting concept is to use a color reference chart in order to stabilize color variations caused by built-in automatic image correction operations [30]. Another option would be adjusting the white balance of the phones to a standardized value. This may be done by using a reference grey card or by locking the exposure and gain while selecting a preset white balance in a control window that is illuminated with an external phone-independent constant light source [32]. Setting the white balance, gain, and exposure can be done in various manual camera applications (e.g., Open Camera, ProShot) and using such a system in combination with a greycard or external constant light source (flashlights of various phones can be different in light output) may decrease inter-phone variation. Moreover, higher sensitivity may be reached by adjusting the exposure time of the assay in such manual camera applications as was previously shown for other smartphone-based LFA quantification for the detection of bacterial fruit blotch [33]. However, the four-parameter dose-response curves did overlap for all four phones for pH determination of buffered soil samples using ΔRGB (Figure 12c) and R (Figure 12d) using the Xiaomi, Huawei, iPhone, and Tablet (R^2^ > 0.99) showing that the proposed method worked without additional adjustments for this assay. Overlap was slightly less when using ΔRGB values, thus, the R channel was used from hereon for this application. Another two phone models (HTC and Samsung) were included to check the universality of the system. Four-parameter dose-response curves showed again excellent fits (R^2^ > 0.99) and overlap with the other curves (Figure 12d). Finally, MAEs of predictions (n = 15) were calculated for each phone using its own calibration curve or a calibration curve constructed with all phone models except the phone model used to take the images for the predictions (Figure 12d; inset). Two-way ANOVA analyses of this data was significant for the type of curve (*p* < 0.001). MAEs increased significantly when predictions were performed on phone A with calibration curves prepared using phones B–F. However, for all phones except the tablet this increase in MAE was below 3% which explains why post-hoc simple effects within phone models and multiple comparisons was only significant for the tablet (*p* < 0.001). Thus, direct quantification of color change by end-users with the pH strips without the need of phone calibration seems possible if slightly increased MAEs are acceptable.

## 4. Conclusions

The ability of all channels of the RGB, HSV, and LAB (named LAC here) color spaces to quantify color intensity and color change variations was tested in paper based and liquid assays. Channel performance was compared under varying background illumination using various phones. A channel of LAC proved not suitable for color quantification and C channel performance was suboptimal. L channel showed good performance in most systems tested (as did V) but both never outperformed the best RGB channel for a specific test. S performed suboptimal in all systems tested. H channel performed satisfactorily for color change but not for color intensity quantification. Moreover, H channel never outperformed the best RGB channel for a specific test. Overall, R functioned best for color change while B and G worked best for color intensity variation. ΔRGB values did show some robustness towards errors in an individual channel of RGB thus providing more universal applicability. However, ΔRGB stops to perform if an error in an individual channel of the RGB color space is too high. Thus, all RGB channels and ΔRGB should be initially plotted for assay development to determine the optimum channel. However, conversions to LAC or HSV color space are unnecessary. These results are specific for colorimetric assays and the implications might not hold for fluorescence or chemiluminescence-based assays where using luminance values seems a more logical choice. This being said, for such assays the light measured will equally pass the Bayer filter of the phone and is converted to RGB values. Mathematically converting these RGB values to LAB values may lead to similar results as reported here. However, a detailed characterization of the performance of RGB, LAB, and HSV color spaces/channels of such luminance-based assays with various phones and fluorophores is needed to test this assumption. Using a light-shielding box to prevent the error caused by background variation was less efficient than internal background correction if images were not taken in direct sunlight. Thus, use of a box is superfluous for colorimetric analysis. For color intensity variation it was shown that images taken with phone A could not be used on a calibration curve taken with phone B. Magnification did improve this situation but did not completely resolve the problem. Thus, camera calibration, white balance adjustment, and exposure time adjustment should be considered for LFA quantification. For color change quantification (pH determination of buffered soil extracts) calibration curves of the six phone models tested overlapped significantly. Thus, color change quantification by end-users without a light-shielding box or phone specific calibration seems feasible. Future research will focus on reducing prediction error and inter-phone variation for color change and intensity-based assays by combining channels from RGB, HSV, and CieLAB color spaces with machine learning algorithms.

## Figures and Tables

**Table 1:** Function types used to fit data points of the papers with varying GNP, CB, LNP concentration, and pH strips measured in various buffers. R^2^ values for each fit are mentioned. The fits with R^2^ > 0.80 were used as calibration curves in Figure 6. C stands for the B channel of LAB which is called LAC throughout the manuscript to avoid confusion of the B channel of LAB with the B channel of RGB. Sigmoidal stands for four-parameter dose-response.

	GNP		CB		LNP		pH	
Channel	R^2^	Function	R^2^	Function	R^2^	Function	R^2^	Function
**R**	0.8871	Sigmoidal	0.9856	Sigmoidal	0.9847	Two phase decay	0.9795	Sigmoidal
**G**	0.9365	Sigmoidal	0.9859	Sigmoidal	0.9830	Two phase decay	0.8350	Sigmoidal
**B**	0.9383	Sigmoidal	0.9877		0.9818	Two phase decay	0.9531	Sigmoidal
**ΔRGB**	0.9374	Sigmoidal	0.9870	Sigmoidal	0.9867	Two phase decay	0.9958	Sigmoidal
**H**	-	-	0.8862	Sigmoidal	0.6042	Two phase decay	0.9844	Sigmoidal
**S**	0.6895	Sigmoidal	-	-	0.7707	Sigmoidal	0.1878	Sigmoidal
**V**	0.8794	Sigmoidal	0.9836	Sigmoidal	0.9803	Two phase decay	0.9600	Sigmoidal
**L**	0.9330	Sigmoidal	0.9861	Sigmoidal	0.9811	Two phase decay	0.9610	Sigmoidal
**A**	-	-	-	-	-	-	0.7979	Sigmoidal
**C**	-	-	-	-	-	-	0.9941	Sigmoidal

**Table 2:** Slopes and R^2^ scores of log–log regression functions fitted to predictions of GNP, CB, LNP concentrations, and pH values shown in the scatter plots of Figure 6. A scoring system was used to attribute scores to R^2^ and slope values for each channel (see Section 2.8). Scores (Table 3) determined ranks for each color system, as well as total scores per channel. Δ is ΔRGB. Ch is channel.

	GNP			CB			Latex			pH			Total Scores	
Rank	Ch	R^2^	Slope	Ch	R^2^	Slope	Ch	R^2^	Slope	Ch	R^2^	Slope	Ch	Score
1	Δ	0.9173	1.008	B	0.979	0.801	V	0.951	1,012	Δ	0.987	0.974	B	37
2	L	0.9008	0.996	R	0.977	0.814	L	0.949	1,049	R	0.996	0.860	R	36
3	G	0.9164	0.972	G	0.973	0.795	G	0.947	1.028	H	0.977	0.874	Δ	35
4	B	0.8856	0.996	L	0.974	0.658	B	0.944	1.051	V	0.927	1.043	V	34
5	R	0.895	1.168	Δ	0.971	0.677	R	0.927	1.074	B	0.973	1.239	L	34
6	V	0.8678	1.058	V	0.972	0.676	Δ	0.762	1.178	L	0.898	0.603	G	32
7	H	-	-	H	0.462	0.354	H	-	-	G	0.712	0.307	H	12

**Table 3:** Scores of the individual channels in each color system, as well as overall scores for all color systems. Shared scores are in bold.

GNP		CB		LNP		pH		Total	
Channel	Score	Channel	Score	Channel	Score	Channel	Score	Channel	Score
ΔRGB	**12**	B	**13**	V	14	ΔRGB	13	B	37
L	**12**	R	**13**	L	**11**	R	11	R	36
G	10	G	9	G	**11**	H	10	ΔRGB	35
B	9	L	7	B	8	V	9	V	**34**
R	6	ΔRGB	6	R	6	B	7	L	**34**
V	5	V	6	ΔRGB	4	L	4	G	32
H	0	H	2	H	0	G	2	H	12

**Table 4:** Two-way ANOVA on phone model and channel for GNP, CB, LNP, and pH predictions.

GNP			CB		LNP		pH Original	
Source	Var	*p*-Value	Var	*p*-Value	Var	*p*-Value	Var	*p*-Value
Interaction	2.8	0.45	8.7	0.0001	25.7	<0.0001	8.1	0.0058
Channel	2.6	0.10	7.1	<0.0001	15.0	<0.0001	7.8	0.0002
Phone	7.9	<0.0001	5.7	<0.0001	5.2	<0.0001	0.8	0.24

**Table 5:** Two-way ANOVA on background illumination and channel for GNP, and pH predictions.

pH			GNP	
Source	Var	*p*-Value	Var	*p*-Value
Interaction	1.652	0.9893	2.177	0.6248
Channels	13.24	<0.0001	0.6425	0.4002
Light conditions	0.9718	0.3046	39.7	<0.0001

**Table 6:** Analytical parameters of prediction and calibration curves for GNP and TMB solution ELISA. R^2^ and slopes given for prediction curves are from linear regression lines. All calibration curves were prepared using four-parameter dose-response functions. LOD, linear range, and IC_50_ values were interpolated from the normalized calibration curves at 90%, 80%–20%, and 50% for R, G, B, and ΔRGB channels and 10%, 20%–80%, and 50% for spectrometer values, respectively.

	Prediction Curves			Calibration Curves (nM)			
Solution Type	Channel	R^2^	Slope	R^2^	LOD	Linear Range	IC_50_
GNP	G	0.9966	1.086	0.9969	1.5	3.1-12.8	7.4
GNP	B	0.9890	1.089	0.9973	1.8	3.7–16.2	9.0
GNP	∆RGB	0.9094	0.824	0.9890	1.6	4.1–32.7	13.8
GNP	L	0.913	0.8009	0.8947	-	-	-
GNP	spectro	0.9989	1.039	0.9996	7.5	15.3–65.7	39.7
HRP	B	0.9742	0.932	0.9957	0.0025	0.0038–0.012	0.0073
HRP	R	0.6087	0.601	0.829	NA	0.010–0.038	0.027
HRP	G	0.8365	0.506	0.9377	NA	0.0083–0.036	0.023
HRP	∆RGB	0.9099	0.734	0.9804	0.0023	0.0039–0.020	0.010
HRP	L	0.07789	0.08	0.9307	-	-	-
	V	0.532	0.29	0.8992	-	-	-
HRP	spectro	0.9752	1.153	0.9982	0.0034	0.0057–0.021	0.012
